# Editorial: Thoracic Surgery in Cancer: Case Reports 2021

**DOI:** 10.3389/fsurg.2022.886499

**Published:** 2022-04-01

**Authors:** Andrew E. Donaldson, Christopher W. Seder

**Affiliations:** Department of Cardiovascular and Thoracic Surgery, Rush University Medical Center, Chicago, IL, United States

**Keywords:** thoracic tumors, rare thoracic cancers, radical resection, multidisciplinary, ECMO

Rare thoracic pathologies and unusual presentations of more common ones can cause diagnostic and treatment dilemmas that may require a more tailored and multidisciplinary approach. In some cases, a definite preoperative diagnosis cannot be made, and the standard treatments are not as established as they are in common thoracic pathologies. In this issue of *Frontiers*, a series of case reports describe the clinical courses of unique thoracic pathologies and provide valuable insight into how we can approach diagnosis and treatment of unusual cases.

Some thoracic pathologies, especially those that are very uncommon, can cause misdiagnoses preoperatively or elude diagnosis altogether until pathologic examination can be performed. Most of the reports in this issue of *Frontiers* describe unusual presentations of very rare pathologies, benign and malignant. In Xiang's et al. report, after a video-assisted thoracoscopic surgery (VATS) resection of a posterior mediastinal mass presumed to be a schwannoma, final pathology identified epithelioid angiosarcoma arising from a schwannoma. This represents an already-rare malignancy arising in a very unusual location, yet successfully managed with surgery alone. Zhi et al. describe a similar situation, in which an anterior mediastinal tumor, initially thought to be a teratoma, was pathologically diagnosed as an epithelioid hemangioendothelioma. Contrary to a teratoma, diagnosis of this rare angiogenic tumor necessitated adjuvant therapy and a rigorous follow-up schedule, without which the patient's recurrence 6 years later may have gone undetected and untreated.

Identification of a rare pathology does not always imply malignancy, however. Zhang et al. report the first case of a pulmonary benign metastasizing adenomyoma. This mass is thought to be a benign metastasis from uterine adenomyoma, and the authors reasonably draw attention to the patient's personal history of uterine leiomyoma and adenomyoma as risk factors. Six months after resection, the patient had no signs of recurrence; perhaps the patient's history imparts a lifetime risk of developing benign metastasis, which only long-term surveillance could tell. In the report by Hu et al., a mass thought initially to be Castleman's disease or a thymoma was found upon intraoperative inspection to be a left innominate vein aneurysm. The authors demonstrate that VATS is a viable approach for management of mediastinal venous aneurysms. If nothing else, these case reports are prime examples of why it is important to always keep a broad differential when working up thoracic masses.

There are some circumstances, such as cases involving very large or locally advanced malignancies, in which radical resection offers the best chance of cure. Cases such as these are ideally handled in a multidisciplinary fashion and sometimes involve coordination with other surgical specialties other than thoracic surgery (1). Cariboni et al. report on the radical resection of a locally advanced paravertebral undifferentiated pleomorphic sarcoma with involvement of the ninth thoracic vertebra and the aortic wall. The treatment team opted for a surgery-first approach, and the combined efforts of thoracic, vascular, and spine surgeons resulted in a successful two-stage R0 resection. At 22 months postoperatively, the patient remained without evidence of disease, representing a favorable outcome made possible by a carefully crafted, multidisciplinary preoperative plan. Yue et al. describe the management of a pulmonary primitive neuroendodermal tumor, a very rare malignancy found in an equally rare location. The size of the tumor−12 × 11 cm—necessitated pneumonectomy, which the patient tolerated well. Long-term follow up would help determine if radical resection alone can provide equivalent survival compared to surgery plus adjuvant therapy. In some cases, local recurrences are unavoidable though, despite the successful completion of a radical resection. This was the case described by Rastrelli et al., in which the patient had a rapidly growing radiation-induced undifferentiated sarcoma of the thoracic wall. In order to obtain negative margins, resection of the mass resulted in a thoracic wall defect that required extensive reconstruction to repair. Unfortunately, the patient developed an aggressive local recurrence and ultimately passed away. All of these cases highlight the importance of clear and open discussions weighing the risks and benefits of radical resection, including the potential for recurrence.

The use of extracorporeal membrane oxygenation (ECMO) support for patients with advanced thoracic malignancies has been documented but is not a common practice (2). In the final report in this series, Signore et al. describe an uncommon presentation of a very common pathology: adenocarcinoma of the lung. After being placed on veno-venous ECMO for acute respiratory failure refractory to mechanical ventilatory support, the patient is found to have a T_4_N_0_M_0_ mass and undergoes a right inferior bilobectomy. Resection immediately improved the patient's oxygenation, indicating that the tumor had been causing a severe pulmonary shunt, and he was weaned off ECMO 3 days later. Although Stage IIIA non-small cell carcinoma is most often managed with neoadjuvant therapy followed by surgery, the patient's clinical status superseded the traditional standard of care. Although the patient recurred and ultimately died 1 year after surgery, the tailored approach to his care gained him a year of life.

Approaching rare thoracic pathologies and unconventional presentations can pose challenges for providers, whether due to the dearth of treatment recommendations or the extent of surgery that may be required to achieve cure. The reports in this issue focus on these unique clinical scenarios and will hopefully provide guidance to providers who are managing similarly challenging cases.

## Author Contributions

Both authors listed have made a substantial, direct, and intellectual contribution to the work and approved it for publication.

## Conflict of Interest

The authors declare that the research was conducted in the absence of any commercial or financial relationships that could be construed as a potential conflict of interest.

## Publisher's Note

All claims expressed in this article are solely those of the authors and do not necessarily represent those of their affiliated organizations, or those of the publisher, the editors and the reviewers. Any product that may be evaluated in this article, or claim that may be made by its manufacturer, is not guaranteed or endorsed by the publisher.
